# The effectiveness of motivation‐guided PDCA cycle nursing for self‐management ability and outcomes of patients with gestational diabetes mellitus

**DOI:** 10.1002/nop2.1903

**Published:** 2023-07-03

**Authors:** Mingxia Zhao, Haixiang Li, Jing Wang, Limin Chu, Lixia Huang, Hailian Li

**Affiliations:** ^1^ Department of Obstetrics The First Hospital of Hebei Medical University Shijiazhuang China; ^2^ Department of Obstetrics and Gynecology Yulin Second Hospital Yulin China; ^3^ Department of Pediatrics The First Hospital of Hebei Medical University Shijiazhuang China; ^4^ Department of Ward Management The First Hospital of Hebei Medical University Shijiazhuang China

**Keywords:** gestational diabetes mellitus, motivation, nursing, outcomes, self‐management

## Abstract

**Aim:**

To explore the effectiveness of motivation‐guided ‘plan, do, check and action’ cycle nursing for self‐management ability and outcomes of patients with gestational diabetes mellitus (GDM).

**Design:**

A pre‐ and post‐ comparison quasi experimental study.

**Methods:**

Totally 108 pregnant women with GDM diagnosed and delivered in our hospital from January 2020 to April 2021 were included in this study. They were divided into study group (54 cases) and control group (54 cases).

**Results:**

The score of self‐management ability were significantly higher than those of control group (*t*‐test, all *p* < 0.05), as well as themselves before interventions in both groups (*t*‐test, all *p* < 0.05). Besides, scores of anxiety, depression, extraverted stimulus and intraverted stimulus all achieved significant reduction after interventions in study group compared with control one (*t*‐test, all *p* < 0.05), as well as themselves before interventions in both groups (*t*‐test, all *p* < 0.05).

**Patient or Public Contribution:**

No patient or public contribution.

## 1 INTRODUCTION

Gestational diabetes mellitus (GDM) is defined as the abnormal glucose metabolism diagnosed at the first time during pregnancy, which often occurs at the medium or late term during pregnancy. It is a kind of metabolic disorder disease which may be induced by abnormal endocrine in pregnancy (Lende & Rijhsinghani, 2020). Recently, overnutrition of the pregnant women are increasing with the progress and economics and the change of dietary structure, which results in the rising incidence of GDM. Persistent high level glucose may cause embryo paraplasia even death, and a high incidence of abortion as much as 15%–30%. Besides, GDM is often complicated with gestational hypertension, polyhydramnios and premature delivery that threatening pregnant women and foetuses (Saravanan et al., 2020). Since most pregnant women with GDM do not need hospitalization and only seek medical treatment in the acute onset or when blood sugar fluctuates greatly, daily life care and good self‐management ability during pregnancy are crucial to improve blood glucose control and pregnancy outcome (Saravanan et al., 2020).

Due to the unnecessary requirements of hospitalization for GDM, self‐management for glucose control is important for the outcomes of pregnancies (Juan et al., 2020). Nowadays, conventional nursing is mainly monodirectional education by nurses, which leading to unsatisfied blood glucose and adverse events (Yan et al., 2021). Motivation‐guided ‘plan, do, check and action’ (PDCA) cycle nursing formulates specific, scientific and personalized interventions for pregnant women based on their health conditions. In accordance with PDCA methods, nurses analyse the effectiveness and revise the inadequacy of interventions via observation and communication of patients, and improvement on interventions will be done next (Liu, 2021; Ren et al., 2019). However, there is few study on investigation of motivation‐guided PDCA nursing for pregnant women with GDM. The application of motivation‐guided PDCA nursing in gestational hypertension can effectively improve pregnancy outcomes, reduce blood pressure and relieve psychological pressure, but whether it can play a certain role in pregnant women with GDM and thus provide them with self‐management ability remains to be discussed (Liu, 2021).

Thus, this study aimed to explore the effectiveness of motivation‐guided PDCA nursing for self‐management ability and outcomes of pregnant women with GDM.

## 2 PATIENTS AND METHODS

### 2.1 Patients

**FIGURE 1:**
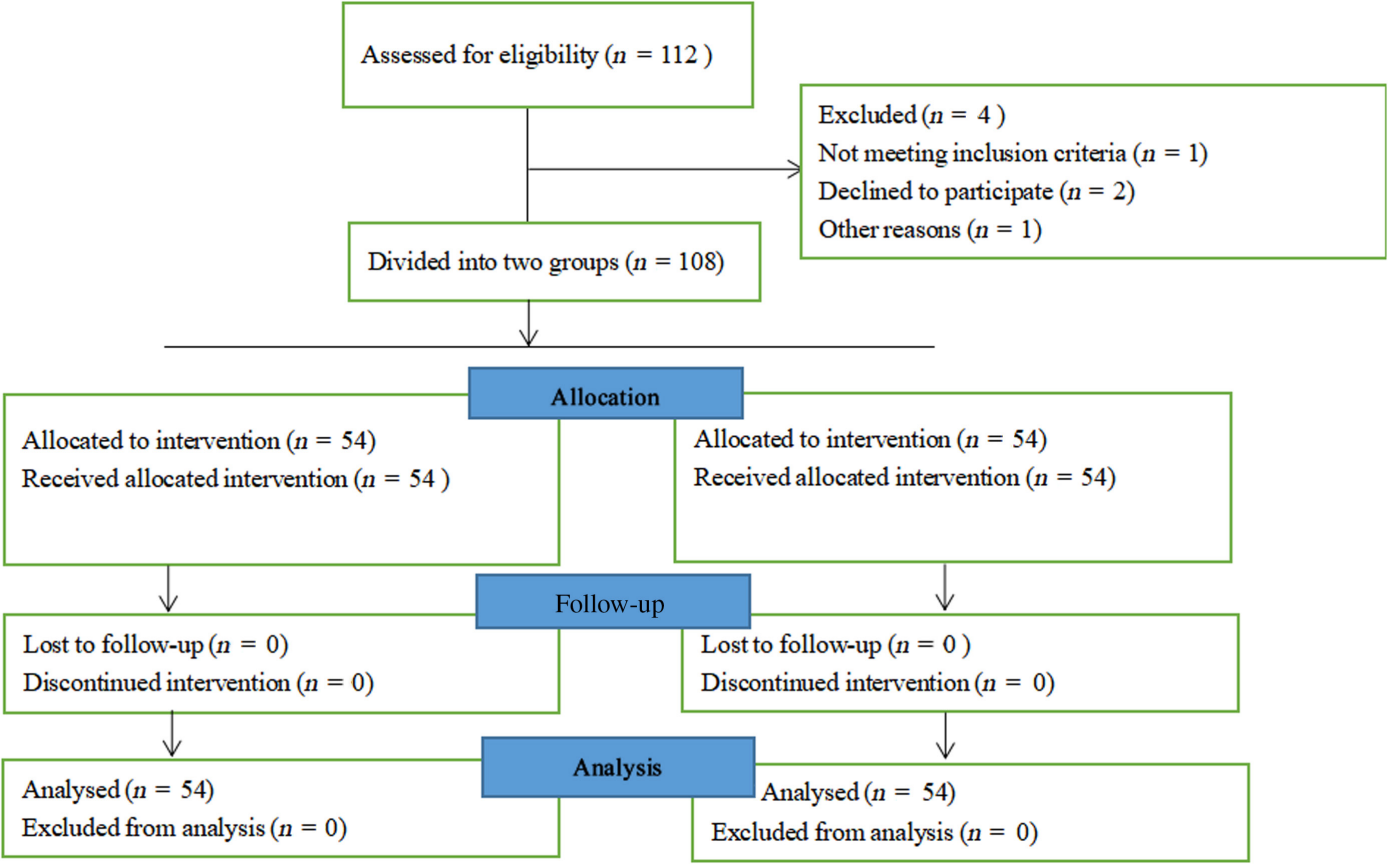
The flow chart of patients.

Pregnant women diagnosed as GDM from January 2020 to April 2021 and finally delivered in our hospital were enrolled into this pre‐ and post‐comparison quasi‐experimental study. In total, 108 patients were allocated to study group (*n* = 54) and control group (*n* = 54). All participants were Chinese female. The flow chart is shown in Figure 1.

The sample size was calculated by the following formula. *N* = [(*Z*_α/2_ + *Z*_β_)σ/δ]^2^(Q1‐1 + Q2‐1; Lv & Feng, 2016). The drop‐out rate was defined as 50%. After calculation, the sample size should be 50. We chose 54 as the sample size.

### 2.2 Inclusion and exclusion criteria

Patients meeting the following criteria were included in this study: (I) diagnosed as GDM by oral glucose tolerance test according to Chinese guideline for prevention and treatment of diabetes (Wang, 2021); (II) routine examination during pregnancy and final delivery in our hospital; (III) patients with natural conception; (IV) with normal mental condition and communication ability. Patients meeting the following criteria were excluded: (I) with primary diabetes before pregnancy; (II) with severe comorbidity conditions; (III) with high risk factors of pregnancy; (IV) with mental disease; (V) with multiple pregnancy; (VI) with coagulation disorders; (VII) allergic to drug used in this analysis; (VIII) with abnormal cardiopulmonary function.

### 2.3 Interventions

Patients in control group were given conventional nursing. Pregnant women were regularly examined by obstetric out‐patient nursing staff and were instructed to attend maternity school run by the hospital after diagnosis. The maternity school provided pregnancy education through the help of slide show and video show in the form of face‐to‐face instruction by doctors, nursing staff and midwives from relevant departments. Family members of pregnant women can accompany them to class. Health guidelines and handbook were given by obstetrics nurses. The handbook contains diabetes related knowledge, diet control, physical exercises, glucose measurement, pharmacy regimens and home health monitoring. Routine health examination were performed every week.

Compared with control group, patients in study group were given addition of motivation‐guided PDCA nursing interventions as follow: (I) plan (P): all treatment strategies were proposed by a PDCA nursing team, including a chief of gynaecology and obstetrics, two obstetricians and gynaecologists, one head nurse, one psychologist, one dietician, two rehabilitation physicians and four nurses of gynaecology and obstetrics. All the members in this team received training for a month and the ones passed the test were enrolled in. The psychologist was in charge of mental health screening, counselling, helping patients to improve cognition of GDM, and reduce uncomfortable feelings and emotions. The personalized nursing plans was made based on the psychological condition of patients. (II) do (D): the nursing team proposed the interventions according to PDCA cycle methods: (a) the nurses in team were in charge of providing health guidelines and handbook, teaching disease‐related knowledge and instructing motivation‐guided PDCA cycle nursing. WeChat was used to sending figures and videos to patients for GDM related knowledge. (b) Team members should instruct the importance and favourable outcomes of actively cooperation during treatment for patients. Also, the confidence of curing disease should be raised. Meanwhile, the team members should confirm the patients' efforts for fighting disease and try to strengthen the positive feedback for disease control. (c) The dietician was in charge of personalized diet plan, sufficient nutrition, balanced dietary structure, various food composition and well glucose control for patients. (d) The rehabilitation physicians were in charge of home exercise plan for patients, helping them select suitable aerobic exercises after meal. Blood pressure and heart rate should recorded after exercises. (e) The self‐introduction videos of pregnant women with GDM with successful delivery were shown to patients under treatment and make an example for them. (f) The team should help patients break the targeted glucose into several small goals in several short periods, make evaluations after each period and supply specific guidelines for the next period. (III) check (C): The head nurse was in charge of checklist for nurses and checking the performance of nurses. All problems in the interventions were summarized and the solutions were discussed every week. (IV) action (A): Deficiencies during the interventions were amended according to feedback of previous acts, and the procedure mentioned above was repeated.

### 2.4 Study index

The glucose level, self‐management ability, psychology status, compliance with health habits and outcomes of patients before and after interventions in both groups were compared.

The glucose level was measured before and after the interventions, including fasting plasma glucose (FPG), postprandial blood glucose (PBG) and glycated haemoglobin (HbAlc). Automatic biochemistry analyser 7180 (Hitachi, Co. Ltd.) was used to analyse the indexes. Glucose oxidase method and enzymic method were used for the test of blood glucose and HbAlc, respectively.

A self‐management questionnaire scale was used for the evaluation of these patients (Li & Li, 2013), including four dimensions, care for foetus, daily life, self‐protection and compliance with medical advice. Each dimension was graded as 0–100. A higher score of this questionnaire indicates greater ability of self‐management. The reliability of the scale is 0.957, Cronbach's alpha is 0.926. The content validity index (CVI) is 0.907.

Irritation, depression and anxiety (IDA) (Yuan et al., 2004) questionnaire scale was used for evaluation of psychology status in both groups, including four dimensions, extraverted stimulus score, intraverted stimulus score, anxiety score and depression score. There are 18 items in total. Each item is graded on a scale of 0–3, among which 6 items (Item 1, Item 2, Item 5, Item 12, Item 13, Item 17) are forward scoring, and the remaining 12 items are reverse scoring. A higher score of this questionnaire indicates more serious irritation, depression and anxiety. The reliability of the scale is 0.957, Cronbach's alpha is 0.769, and the CVI is 0.810.

Compliance with medical advice of patients (Li et al., 2020) before and after interventions were recorded in both groups, including four dimensions, self‐management, weight monitoring, exercises and dietary. Each dimension is graded as 0–10. A higher score in this scale indicates a better compliance with medical advice.

Blood loss in 2 h after surgery, gestational hypertension, caesarean delivery, premature delivery, foetal macrosomia, polyhydramnios, neonatal hypoglycemia, foetal distress and Apgar score of foetus were recorded.

All data were collected and analysed by two independent nursing staff. Data would be reviewed by the head nurse in case of discrepancies.

### 2.5 Indications for caesarean delivery

The majority of pregnant women who received C‐section were strictly follow the indications, including foetal distress, previous caesarean section, non‐progress of labour, oligohydramnios, malpresentation, cephalo pelvic disorders, hypertensive disorder in pregnancy (Maskey et al., 2019).

### 2.6 Statistical analysis

All the data collected in this study were analysed using SPSS 21.0 software (IBM Co., Ltd.). Continuous data conforming to normal distribution were expressed as mean (SD), and the comparisons were examined by Student‐*t* test. The categorical data were expressed as *n* (%), and the differences between the two groups were examined by chi‐square analysis or Fisher's exact test. *p* < 0.05 was considered statistically significant.

## 3 RESULTS

### 3.1 Baseline characteristics

There were 32 (59.26%) unipara patients and 22 (40.74%) multipara patients in study group with a mean age of 26.89 (4.08, range 23–36) years and a mean body mass index (BMI) of 27.68 (3.19, range 19.81–32.23) kg/m^2^. The mean gestation week of these patients were 23.32 (2.87, range 20–25) weeks. The mean number of pregnancies was 3.38 (1.09, range 1–4) times and the mean number of foetus deliveries was 1.67 (0.41, range 1–3) times. There were 33 (61.11%) unipara patients and 21 (38.89%) multipara patients in control group with a mean age of 26.91 (4.12, range 23–36) years and a mean BMI of 27.72 (3.22, range 19.83–32.28) kg/m^2^. The mean gestation week of these patients were 20.36 (2.79, range 20–25) weeks. The mean number of pregnancies was 3.35 (1.07, range 1–4) times and the mean number of foetus deliveries was 1.71 (0.43, range 1–3) times. All these baseline characteristic between 2 groups were with no significant difference (*t*‐test or *χ* ^2^ test, all *p* > 0.05).

### 3.2 Study index

There was no difference of FPG, PBG and HbAlc between these 2 groups before interventions (*t*‐test, all *p* > 0.05) but both with a significantly decrease after interventions (*t*‐test, all *p* < 0.05). Besides, the indexes in study group were significantly lower than those of control group after interventions (*t*‐test, all *p* < 0.05).

There was no difference of self‐management scores between the study and the control group before interventions (*t*‐test, all *p* > 0.05). After interventions, the scores were both significantly increased compared with before in both groups (*t*‐test, all *p* < 0.05). In addition, the scores after interventions in study group were significantly higher than those of control group (*t*‐test, all *p* < 0.05).

There was no difference of IDA scores between these 2 groups before interventions (*t*‐test, all *p* > 0.05). However, significant reduction of scores were obtained in both groups after interventions (*t*‐test, all *p* < 0.05). Furthermore, the scores after interventions in study group were significantly lower than those of control group (*t*‐test, all *p* < 0.05).

There was no difference of compliance scores between these 2 groups before interventions (*t*‐test, all *p* > 0.05). Nevertheless, scores were significantly increased in both groups after interventions (*t*‐test, all *p* < 0.05). Furthermore, the scores after interventions in study group were significantly higher than those of control group (*t*‐test, all *p* < 0.05).

All results mention above could be seen in Table 1.

**TABLE 1:** Comparison of glucose, self‐management ability, psychological score and compliance with medical advice of patients included.

Variables	Study group (*n* = 54)	Control group (*n* = 54)	*t*‐value	*p*‐Value
Glycated haemoglobin (%), mean (SD)				
Before intervention	9.32 (1.31)	9.38 (1.28)	−0.241	0.809
After intervention	6.51 (1.23)*	7.92 (1.19)*	−6.054	<0.001
Postprandial blood glucose (mmol/L), mean (SD)				
Before intervention	10.17 (1.34)	10.12 (1.32)	0.208	0.836
After intervention	7.42 (1.18)*	8.47 (1.09)*	−4.803	<0.001
Fasting plasma glucose (mmol/L), mean (SD)				
Before intervention	7.45 (1.09)	7.42 (1.07)	0.144	0.886
After intervention	4.56 (1.12)*	6.09 (1.18)*	−6.911	<0.001
Care for fetus, mean (SD)				
Before intervention	63.23 (2.19)	63.31 (2.23)	−0.188	0.851
After intervention	92.19 (3.29)*	82.16 (3.32)*	15.986	<0.001
Daily life, mean (SD)				
Before intervention	65.28 (2.27)	65.31 (2.31)	−0.068	0.946
After intervention	87.98 (2.38)*	79.81 (2.41)*	17.725	<0.001
Self protection				
Before intervention	63.18 (4.39)	63.23 (4.52)	−0.058	0.954
After intervention	86.92 (5.09)*	78.98 (5.15)*	8.058	<0.001
Compliance with medical advice				
Before intervention	62.18 (5.29)	62.23 (5.23)	−0.049	0.961
After intervention	90.98 (5.27)*	85.29 (5.32)*	5.584	<0.001
Extraverted stimulus score				
Before intervention	13.29 (1.34)	13.24 (1.32)	0.196	0.846
After intervention	4.03 (1.02)*	6.03 (1.05)*	−9.739	<0.001
Intraverted stimulus score				
Before intervention	12.32 (1.32)	12.28 (1.27)	0.159	0.873
After intervention	4.21 (1.01)*	5.93 (1.03)*	−8.762	<0.001
Anxiety score				
Before intervention	13.29 (1.28)	13.23 (1.24)	0.247	0.805
After intervention	6.73 (1.02)*	8.93 (1.03)*	−11.153	<0.001
Depression score				
Before intervention	14.02 (1.23)	13.97 (1.19)	0.215	0.827
After intervention	6.03 (1.04)*	8.76 (1.13)*	−13.063	<0.001
Compliance with self‐management				
Before intervention	3.29 (0.23)	3.31 (0.27)	−0.414	0.679
After intervention	8.32 (0.32)*	7.34 (0.35)*	15.185	<0.001
Compliance with weight monitoring				
Before intervention	3.32 (0.25)	3.34 (0.28)	−0.392	0.696
After intervention	8.38 (0.28)*	7.31 (0.29)*	19.505	<0.001
Compliance with exercises				
Before intervention	3.37 (0.28)	3.39 (0.29)	−0.365	0.716
After intervention	8.41 (0.32)*	7.38 (0.23)*	19.207	<0.001
Compliance with dietary				
Before intervention	3.32 (0.26)	3.38 (0.28)	−1.154	0.251
After intervention	8.35 (0.35)*	7.33 (0.29)*	16.489	<0.001

* *p* < 0.05 compared with those before interventions in the same group.

The blood loss in 2 h after surgery were significantly reduced in study group than that of control one (*t*‐test, all *p* < 0.05). Besides, the incidence of caesarean delivery, premature delivery, foetal macrosomia, polyhydramnios and neonatal hypoglycemia were significantly lower than those of control group (*χ* ^2^ test, all *p* < 0.05). The incidence of gestational hypertension and foetal distress were with no difference between 2 groups (*χ* ^2^ test, both *p* > 0.05). Moreover, there was also no difference of neonatal Apgar score between these 2 groups (*t*‐test, *p* > 0.05; Table 2).

**TABLE 2:** Comparison of delivery outcomes.

Variables	Study group (*n* = 54)	Control group (*n* = 54)	*t*/*χ* ^2^ value	*p*‐Value
Blood loss in 2 h after surgery (mL), mean (SD)	209.89 (28.29)	281.29 (29.73)	−12.785	<0.001
Gestational hypertension, *n* (%)	1 (1.85%)	4 (7.41%)	1.887	0.169
Caesarean delivery, *n* (%)	8 (14.81%)	21 (38.89%)	7.967	0.005
Premature delivery, *n* (%)	1 (1.85%)	7 (12.96%)	4.859	0.027
Foetal macrosomia, *n* (%)	0 (0.00%)	7 (12.96%)	7.485	0.006
Polyhydramnios, *n* (%)	0 (0.00%)	7 (12.96%)	7.485	0.006
Neonatal hypoglycemia, *n* (%)	0 (0.00%)	6 (11.11%)	6.353	0.012
Foetal distress, *n* (%)	0 (0.00%)	1 (1.85%)	1.009	0.315
Apgar score of foetuses, mean (SD)	96.39 (1.23)	96.23 (1.02)	0.736	0.463

## 4 DISCUSSION

Previous studies have shown that the incidence rate was 1%–14% in China (Han et al., 2019). With the change of life style and the comprehensive opening of two children policy in China, there are more and more elderly pregnant women with high risk of pregnancy, including GDM. GDM is not only harmful to pregnant women, foetuses and newborns but also increases the risk of developing into diabetes post‐delivery. About 50% of pregnant women with GDM may develop into diabetes even after delivery, which will bring burdens to society. Therefore, early interventions and improvement of self‐management ability is important for effective prevention and outcomes of these patients (Alejandro et al., 2020).

PDCA cycle quality management method has been widely used in clinical practice to promote empirical nursing transforming to scientific nursing and raise nursing quality (Liu et al., 2022). In this study, motivation was implemented as guidelines and combined with PDCA method to set successful example for self‐management strengthen, psychology status improving and glucose control of patients. Ma et al. (2020) showed that effective nursing interventions could well control glucose level, highly strengthen self‐management ability and favourably improve outcomes of pregnant women with GDM. Wang et al. (2020) proposed that active nursing interventions could control glucose, raise compliance and improve outcomes of these patients. Present study confirmed the evidence mentioned above. A significant reduction of FPG, PBG and HbAlc after interventions showed the effectiveness of PDCA cycle nursing on glucose control. It might be induced by the favourable relationship between patients and nurse, which would promote patients change life style, pay attention to self‐management and monitor health conditions according to personalized health care plans.

Self‐management ability is a new health management model, which has been widely used. It can play an important role in the management of pregnant women with GDM (Zhang et al., 2020). Good self‐management behaviour can promote patients to accept new concept of health, enable them to develop healthy behaviour, enhance the awareness of active participation and effectively improve blood glucose and outcomes (Zhang et al., 2020). Liang and Jin (2021) proposed that the self‐management behaviour of pregnant women with GDM is not ideal at present. Effective interventions should be taken among these patients. Jia et al. (2020) showed that effective nursing interventions can improve the self‐management ability of pregnant women with GDM. In present study, the scores of care for foetal, daily life, self‐protection and compliance with medical advice in the study group were significantly higher than those in control group after interventions (*p* < 0.05). It reminded us that motivation‐guided PDCA cycle nursing could effectively improve the self‐management ability of pregnant women with GDM. Conventional nursing interventions is actively guided by nurses and passively accepted by patients, which is difficult to motivate patients themselves (Zhang et al., 2020). Motivation‐guided PDCA cycle nursing can enhance the self‐management ability of pregnant women with GDM by the following measures. First, setting up PDCA cycle nursing team to make strict implementation of nursing plan. Second, in the implementation process, patients were constantly encouraged to correct behaviour, combined with dietary intervention. Third, combined with family exercise intervention and rehabilitation goals, it can promote patients to realize the influence of healthy lifestyle on GDM.

All pregnant women with GDM have great negative psychology. Most patients are worried about the health conditions of foetus and themselves. The feelings of depression and anxiety could be resolved after effective interventions (Liu, 2020). Duan et al. (2021) proposed that effective nursing interventions could control the blood glucose level, improve their psychology status and delivery outcomes of pregnant women with GDM, which was worth to be widely used. Present study showed that scores of extraverted stimulus, intraverted stimulus, anxiety and depression in study group were significantly lower than those of control group after interventions. It reminded us that motivation‐guided PDCA cycle nursing could effectively improve the psychology status of pregnant women with GDM. By explaining to patients the contents, methods and effects of PDCA cycle nursing, nursing staff can establish trust relationship with patients and reduce the psychological pressure of patients.

Better compliance with medical advice may lead to favourable outcomes. Previous study has shown favourable blood glucose control and delivery outcomes in high pregnant women with GDM highly complied with medial advice (O'Dea et al., 2014). However, most of pregnant women with GDM were actually with poor compliance due to life, work and other reasons during pregnancy. Previous study (Zhang et al., 2019) showed that effective nursing interventions for pregnant women with GDM could improve the cognition of disease, raise their compliance, and reduce adverse events during pregnancy and delivery. This study showed that scores of self‐protection, weight monitoring, exercises and dietary behaviour in the study group were significantly lower than those of control group after the interventions. This reminded us that motivation‐guided PDCA cycle nursing could effectively improve the compliance of health behaviour in pregnant women with GDM. This nursing method explained the hazards and prevention of GDM to the patients via pictures and videos and promote more comprehensive understanding of disease related knowledge in these patients. After understanding the harm of this diseases to maternal and infant health, patients can take the initiative to carry out nursing work and improve compliance with healthy behaviours during pregnancy.

Previous study has shown that conventional nursing interventions were easy to result in high incidence of adverse event during pregnancy (Wei, 2021), because of the lack of specific nursing interventions (Wang et al., 2020). Lin et al. (2021) found that the incidence of adverse events in pregnant women with GDM was high and should be paid more attention to prevent. Gong and Lin (2020) found that effective nursing interventions for pregnant women with GDM could improve delivery outcomes and ensure patients health conditions. This study showed that the blood loss 2 h after surgery, caesarean delivery, premature delivery, foetal macrosomia, polyhydramnios and neonatal hypoglycemia in study group were significantly lower than those of control group. It reminded us that motivation‐guided PDCA cycle nursing could improve delivery outcomes of pregnant women with GDM. It might be due to repeated revision and promotion of PDCA cycle nursing for patients. Furthermore, nursing stuff continuously improved the nursing plan, carried out the whole process management, effectively control the weight and blood sugar level. The above measures reduced the occurrence of pregnancy complications, thus to improve the pregnancy outcome.

There were also several limitations in this study. First, there were unavoidable biases due to its non‐prospective nature of this study. Second, this was a single centre study with small sample size. Thus, all results and conclusions should be interpreted cautiously. More prospective studies with large sample size should be made in the future.

## 5 CONCLUSION

In conclusion, motivation‐guided PDCA cycle nursing could effectively control blood glucose, strengthen self‐management ability, relief psychology status, raise compliance with medical advice and improve delivery outcomes of pregnant women with GDM.

## AUTHOR CONTRIBUTIONS

Mingxia Zhao and Hailian Li contributed to the conception and design of the study; Haixiang Li, Limin Chu and Jing Wang performed the experiments, collected and analysed data; Lixia Huang, Mingxia Zhao and Hailian Li wrote the manuscript; Mingxia Zhao and Hailian Li revised the manuscript. All authors reviewed and approved the final version of the manuscript.

## FUNDING INFORMATION

This study was funded by Medical Science Research Project of Hebei Provincial Health Commission (No. 20221438).

## CONFLICT OF INTEREST STATEMENT

The authors declare that they have no competing interests.

## ETHICS STATEMENT

This study was approved by the Ethics Committee of The First Hospital of Hebei Medical University (No. 20200624). Informed consent was obtained from all the study subjects before enrollment.

## CONSENT FOR PUBLICATION

Not applicable.

## Data Availability

The datasets generated and analysed during the current study are available from the corresponding author on reasonable request.
